# Epidemiology of Vasculitides in Khorasan Province, Iran

**Published:** 2015-07

**Authors:** Mohammadhassan Jokar, Zahra Mirfeizi

**Affiliations:** Department of Internal Medicine, Imam Reza Hospital, Mashhad University of Medical Sciences, Mashhad, Iran

**Keywords:** Vasculitis, Systemic vasculitis, Vascular diseases, Arteritis

## Abstract

Vasculitides are a heterogeneous group of more than 20 diseases defined by inflammation and destruction of blood vessels. We aimed to study the demographic characteristics of the primary vasculitides in the North East of Iran. We retrospectively studied the medical records of patients diagnosed with any kind of vasculitis at the Clinic and Department of Rheumatology of the Imam Reza Hospital, Mashhad, Iran between January 1, 2002, and December 31, 2012. Patients were classified according to the American College of Rheumatology 1990 criteria for the classification of vasculitis and the 2012 Revised International Chapel Hill Consensus Conference Nomenclature of Vasculitides. A total of 721 patients (51.5% male, 48.5% female) with a diagnosis of primary vasculitis was identified. The frequency distributions of vasculitic disorders were as follows: Behcet’s disease, 63.6%; cutaneous leukocytoclastic angiitis, 8.2%; granulomatosis with polyangiitis (Wegener’s), 6.8%; Takayasu’s arteritis. 6%; giant cell arteritis, 4%; polyarteritis nodosa, 2.1%; microscopic polyangiitis, 0.6%; eosinophilic granulomatosis with polyangiitis (Churg-Strauss), 1.8%; cryoglobulinemic vasculitis, 0.3%; and IgA vasculitis (Henoch-Schonlein purpura), 3.5%. In our population, the most common forms of vasculitis are Behcet’s disease, cutaneous leukocytoclastic angiitis, and granulomatosis with polyangiitis (Wegener’s).

## Introduction

Vasculitides are a heterogeneous group of more than 20 diseases defined by the presence of inflammatory leukocytes in blood vessel walls. Reactive damage to mural structures with eventual loss of vessel integrity can lead to bleeding. Inflammation of the vessel wall leads to thickening and/or fibrosis, with subsequent obliteration of its lumen. Numerous clinical syndromes associated with systemic symptoms (e.g., fever, weight loss, anorexia) result from ischemia of tissues supplied by the affected vessels.The vasculitides are sometimes serious and fatal diseases that require immediate recognition and therapy.^1^

There is a wide variety of vasculitic syndromes, grouped according to the size of the affected vessels and by the dominant clinical pattern. Recently the presence or absence of antineutrophil cytoplasmic antibodies (ANCA) has been added to proposed classification criteria. New nomenclature for the various forms of systemic vasculitis was published in January 2013.^2^ This system of naming vasculitis updates the nomenclature published in 1994 by the first International Chapel Hill Consensus Conference on the Nomenclature of Systemic Vasculitides.^3^While providing useful definitions of vasculitides, the conference was not expected to formulate classification or diagnostic criteria.

Vasculitis may occur as a primary process or may be secondary to another underlying disease (e.g., other connective tissue diseases, drugs, malignancies, or infections).^4^ The primary vasculitides (PV) are a group of conditions characterized by inflammation and necrosis of blood vessel walls. The etiology of these conditions is unknown, but geographic, environmental, and genetic factors are important to causality. Until recently, there were relatively few accurate, descriptive epidemiologic data available.^5^

The demographic elements of vasculitic disorders have never been studied in Iran. Here, we present for the first time the demographic features of primary vasculitides in the population of Iran. 

## Patients and Methods

We retrospectively studied the medical records of our patients who were diagnosed with any type of primary vasculitis at the Department and Clinic of Rheumatology of Imam Reza Hospital, Mashhad, Iran between January 1, 2002, and December 31, 2012. Patients were classified according to the American College of Rheumatology (ACR) 1990 vasculitis criteria^6^ and 2012 Revised International Chapel Hill Consensus Conference Nomenclature.^2^ All inpatients and outpatients who met the ACR 1990 vasculitis criteria were included. Patients with secondary vasculitis were excluded from the study.

*Statistical Analysis*

Continuous data were shown as means and standard deviations (mean±SD), and categorical variables as percentages. 

## Results

A total of 721 patients (51.5% male, 48.5% female) diagnosed with primary vasculitis (PV) were identified between January 1, 2002 and December 31, 2012. The overall frequency of PV was nearly equal between males and females (male/female ratio of 0.98 to 1). Table 1 shows the frequency distribution by PV type, percentage of female population with PV, age, and frequency proportion for each diagnostic category.

**Table 1:** The frequency distribution of vasculitic disorders in the North East of Iran

**Type**			**Female %**	**Age (years)**			**Frequency no. (%)**
				**Minimum**	**Maximum**	**Mean**	
LVV	Total		76.4	17	83	42.9	72 (10.0)
	TAK		94.7	17	48	27	43 (6)
	GCA		48.3	45	83	64	29 (4)
MVV	Total		53.3	11	82	41.4	15 (2.1)
	PAN		53.3	11	82	41	15 (2.1)
	KD		0	-	-	-	0 (0)
SVV	Total		48.4	5	86	34	153 (33.3)
	AAV	Total	51.5	11	66	35.9	66 (9.2)
		GPA	55.1	11	61	33	49 (6.8)
		EGPA	30.8	23	66	50	13 (1.8)
		MPA	75	19	35	27	4 (0.6)
	ICV	Total	48.1	5	54	21.4	27 (3.8)
		AGBM	-	-	-	-	0 (0)
		CV	100	35	43	39	2 (0.3)
		IGAV	44	5	54	20	25 (3.5)
		HUV	-	-	-	-	0 (0)
VVV	Total		48.4	11	78	33.9	477 (66.2)
	BD		48.6	11	78	33.9	477 (66.2)
	CS		-	-	-	-	0 (0)
SOV	Total		50	5	86	34.5	4 (0.6)
	CLA		45	11	86	38	60 (8.2)
	CA		-	-	-	-	0 (0)
	PCNSV		-	-	-	-	0 (0)
	IA		-	-	-	-	0 (0)
	OTHERs		51.5	18	30	24	4 (0.6)
Total			51.5	5	86	34.54	721 (100)

LVV: Large vessel vasculitis; TAK: Takayasu arteritis; GCA: Giant cell arteritis; Medium vessel vasculitis; PAN: Polyarteritis nodosa; KD: Kawasaki disease; SVV: Small vessel vasculitis; AAV: Antineutrophil cytoplasmic antibody (ANCA)-associated vasculitis; MPA: Microscopic polyangiitis; GPA: Granulomatosis with polyangiitis (Wegener’s); EGPA: Eosinophilic granulomatosis with polyangiitis (Churg-Strauss); ICV: Immune complex; SVV: Small vessel vasculitis; AGBM: Anti-glomerular basement membrane disease; CV: Cryoglobulinemic vasculitis; IGAV: IgA vasculitis (Henoch-Schonlein); HUV: Hypocomplementemic urticarial vasculitis; VVV: Variable vessel vasculitis; BD: Behcet’s disease; CS: Cogan’s syndrome; SOV: Single-organ vasculitis; CLA: Cutaneous leukocytoclastic angiitis; CA: Cutaneous arteritis; PCNSV: Primary central nervous system vasculitis; IA: Isolated aortitis

## Discussion

Vasculitis refers to a heterogeneous group of disorders that is characterized by inflammatory destruction of blood vessels. Inflamed blood vessels can be occluded or ruptured, and thereby lose its ability to deliver oxygen and other nutrients to tissues and organs. Depending on the severity, distribution, and size of the affected blood vessels, vasculitis can result in clinical syndromes that vary in severity from a self-limited skin rash to a life-threatening multisystem disease.^1^

Because it often begins with constitutional and nonspecific symptoms and signs and unfolds slowly over weeks or months, vasculitis is one of the greatest diagnostic challenges in medicine. Establishing the diagnosis of vasculitis requires confirmation by laboratory tests, usually a biopsy of an involved tissue and sometimes an angiogram.^4^^,^^5^

The demographic and epidemiologic characteristics of vasculitic disorders vary greatly by geography. This variation may reflect genetics, environmental differences, and the prevalence of other risk factors.^7^

Although some patients with vasculitis are seen by other specialists (dermatologists, pediatricians, internists), only patients who were seen and managed by a rheumatologist were recruited in our study. Thus, some forms of vasculitis (e.g. IgA vasculitis, cutaneous leukocytoclastic angiitis, and Kawasaki’s disease) may be underestimated.

Vasculitis can be as a primary process or may be secondary to another underlying disease.^4^ We studied only patients with primary vasculitis; patients with secondary vasculitis were not included.

A total of 721 cases comprised this study. Table 1 shows the frequency distribution of vasculitic disorders in our patients. For comparison, the frequency distributions of vasculitic disorders in three other studies are shown too (table 2).^6^^,^^8^^,^^9^

**Table 2:** Frequency distribution of types of vasculitis in our study and three other studies

**Vasculitis**	**Iran** **(%)**	** ACR^6^** **(%)**	** India^8^** **(%)**	** Denmark^9^** **(%)**
Takayasu’s arteritis	6.3	6	20.20	1
Giant cell arteritis	4	22.3	3.36	14.4
Polyarteritis nodosa	2.1	12.3	8.83	2
Wegener’s granulomatosis	6.8	8.7	13.81	27.8
Churg-Strauss syndrome	1.8	2.1	1.7	2
Microscopic polyangiitis	0.6	-	3.9	12.3
IgA vasculitis (Henoch-Schonlein)	3.5	8.7	21.8	2
Kawasaki’s disease	0	5.4	0.4	0
Behcet’s disease	63.3	0	13.6	0
Cryoglobulinemic vasculitis	0.3	0	0	0
Cutaneous leucocytoclastic angiitis	8.2	-	-	38.1

The most common form of vasculitis in our study was Behcet’s disease (BD). About 63% of our patients diagnosed with vasculitis had BD. Behcet’s disease is named after the Turkish dermatologist who first described the syndrome in 1937 as a triad of recurrent oral ulcers, genital ulcers, and eye inflammation. Although these clinical findings are often the most salient, BD can involve almost any organ. Although this disease is seen worldwide, the prevalence is highest in countries of the Eastern Mediterranean, the Middle East, and East Asia.^10^ The prevalence of BD in Iran is 8 per 10,000 inhabitants,^11^ the second highest prevalence after Turkey (8-37 per 10,000).^12^ BD is a disease of young adults; typically, patients are in their 20s or 30s when symptoms first develop. Although men are more commonly affected than women in the Middle Eastern countries, but in Japan women more commonly involved.^10^ In our study, the mean age of patients with BD was 33.9 years that was more than Davatchi’s study (26 years).^13^ Male to female ratio in our patients was 1.06 that was less than Davatchi’s study (male/female ratio 1.22).

Takayasu’s arteritis, named after the Japanese ophthalmologist who first described the ocular manifestations in 1908, is a large-vessel vasculitis of unknown etiology that mainly affects women during their reproductive years. It can be seen all over the world, with the greatest prevalence in Asians. The male:female ratio has been reported from 1:9 in the Japanese population to 1.3:1 in the Indian population. The mean age of onset is usually between 10 and 40 years. The frequency of Takayasu’s arteritis among our patients was 6%, which was the same as that reported by the American College of Rheumatology.^6^ In our study the male:female ratio was 1:7.6.^14^

Giant cell arteritis (GCA), also known as temporal arteritis, occurs almost exclusively in older people and mainly affects the extracranial branches of the carotid artery. GCA in our study was as frequent as the Indian study,^8^ but considerably lower than that of western countries.^8^^,^^9^ Although the literature reports that more women are affected by giant cell arteritis than men, our study did not disclose this gender preference. In our study, the male to female ratio was 1.1 to 1, which correlated with other studies on the Asian population.^15^ Although it has been reported in individual patients younger than 50 years, GCA is usually encountered in the elderly. The mean age at onset is 72 years, with a range from approximately 50 to older than 90 years. In our study, the mean age of onset was slightly lower (63.97 years). Only one patient was younger than 50. Compared with the Caucasian population, this incidence pattern of a slightly younger age group was also observed in other case studies from Asia.^15^

Classic Polyarteritis nodosa (PAN) is a multisystem disorder characterized by necrotizing inflammation of small or medium arteries that usually spares the smallest blood vessels (e.g., arterioles or capillaries) and is not associated with glomerulonephritis. PAN can be seen at any age, but the most common age range at diagnosis is 40 to 60 years. There is no clear gender difference.^16^ PAN in our study was as frequent as in a study from Denmark^9^ (2.3% and 2%, respectively), but lower than that of ACR^6^ and India.^8^ In our study the male to female ratio was 0.88 to 1 and the mean age was 41 years.

Antineutrophil cytoplasmic antibodies (ANCA) associated vasculitis (AAV) comprise a group of three heterogeneous syndromes: granulomatosis with polyangiitis (Wegener’s), microscopic polyangiitis (MPA), and eosinophilic granulomatosis with polyangiitis (Churg–Strauss).^16^ In our patients, the frequencies of granulomatosis with polyangiitis (Wegener’s) and eosinophilic granulomatosis with polyangiitis (Churg–Strauss) were correlated with that of ACR study (table 2). Microscopic polyangiitis was very rare among our patients.

IgA vasculitis (Henoch-Schonlein) is the most common form of systemic vasculitis in children.^17^ Ninety percent of cases are in the pediatric age group. There is a male predominance with reported male-to-female ratios of 1.2:1 to 1.8:1.^18^ IgA vasculitis in our study was as frequent as ACR study,^8^ but much lower than Denmark study. In our study, it was slightly more common in males (1.2:1).

Cutaneous leukocytoclastic angiitisis (hypersensitivity vasculitis) is a clinical term that generally refers to an Immune Complex-mediated small vessel vasculitis of the skin that spares internal organs and usually follows drug exposures or infections.^19^ It was the second most common type of vasculitis in our study (8.2%). The frequency of Cutaneous leukocytoclastic angiitisis in the population of Denmark^10^is much higher than the population of the northeast of Iran (table 2). We found a male:female ratio of 0.8 to 1 and the mean age was 38 years.

## Conclusion

The most common forms of vasculitis in the northeast of Iran are Behcet’s disease, cutaneous leukocytoclastic vasculitis, and granulomatosis with polyangiitis (Wegener’s). 
